# Establishing a nurturing micro-sleep environment may positively influence children's overall development^☆^

**DOI:** 10.1016/j.sleepx.2025.100162

**Published:** 2025-11-19

**Authors:** Oliviero Bruni

**Affiliations:** Department of Human Neuroscience, Sapienza University, Rome, Italy

## 1 Introduction

The Special Issue “Sleep Without Borders” arrives at a moment when the field is actively reframing sleep as a population health imperative rather than a purely clinical endpoint [1]. In this context, Will-Dryden and colleagues’ report from Tanzania offers a quietly radical proposition: that constructing a safe, climate-appropriate micro-sleep environment—a mattress, a net, basic bedding, and culturally tuned education—can be a scalable lever for child development in low-resource settings [2]. Over 24 distributions from 2000 to 2024, the program delivered 135,500 bed kits to children aged 7–11; mattresses were paired with insecticide-treated nets in every cohort, and since 2019 brief, orally delivered, parent-focused sleep education has been integrated into the effort [2].

A modest “micro-environment,” such as a safe, climate-appropriate place to sleep with a basic mattress, net, and bedding, can play a significant role in influencing neurodevelopmental risk, especially in low-resource settings. By directly addressing material scarcity through the provision of essential items like a mattress and sheets, and by offering vector protection with insecticide-treated nets, these environments can reduce the physical and biological stressors that often disrupt children's sleep.

Development hinges on sleep continuity—and in childhood, sleep fragmentation rather than mere duration often carries the heaviest toll for attention, memory consolidation, and executive function [[3], [4], [5], [6], [7]]. Environments marked by overcrowding, heat, noise, and vector exposure undermine continuity through repeated arousals, anxiety, and illness [8]. In malaria-endemic regions, the perceived and actual threat of mosquito bites can induce hypervigilance, which underlies insomnia, while malaria itself is not only difficult to endure but also further disrupts sleep and school attendance [9]. Providing a supportive sleep surface, a barrier against mosquito bites, and comfortable bedding may help target the proximate determinants of arousal and nocturnal threat. This is expected to improve sleep health and set the stage for an enhanced next-day behavior and learning [[3], [4], [5], [6], [7]].

Altogether, creating a supportive micro-sleep environment can have downstream effects on child development, potentially improving sleep quality, school readiness, and overall well-being.

The Tanzanian program maps neatly onto this causal pathway. It addresses material scarcity (mattress, sheets) and vector protection (insecticide-treated net); it uses schools as delivery hubs, and it adds brief parent education adapted for low literacy—an approach likely to strengthen routines and expectations around bedtime, further buffering fragmentation [2,10,11].

One notable aspect of this work is its community-centered “push” approach: local partners select recipients and collaborate with ministries and school leaders to develop content. The initiative moves forward even as data systems are still being developed. The authors contrast this with a “pull” paradigm in which programs await definitive trials before deployment. In settings where the counterfactual is no bed, no net, and no instruction, the push model is ethically defensible and pragmatically necessary—and, crucially, it can generate the very implementation data that later underwrite more rigorous evaluation [12]. This stance also challenges a common blind spot: the uncritical generalization of sleep science from high-income contexts to households where the meaning of “bedroom,” “bed,” and “quiet” diverges profoundly; advancing sleep health equity requires solutions matched to local ecologies and economies, not only to communities that can afford the luxury of polysomnographic standards [13].

Three features deserve emphasis for policy and practice: (1) Two dozen distributions over nearly a quarter century, with mosquito nets and mattresses in every kit, create a stable platform for measuring downstream effects on sleep and school readiness [2]. (2) Schools provide reach, logistics, and legitimacy; they are natural conduits for brief, culturally tailored sleep education to caregivers—here, delivered orally to fit literacy realities [10,11,14]. (3) Early inclusion of pillows was discontinued when found culturally incongruent, illustrating iterative tailoring rather than one-size-fits-all provision [2].

The authors demonstrate appropriate transparency by acknowledging that empirical sleep and daytime outcomes were not obtained, and that program data primarily originate from archival operational records [2]. Future studies should maintain the community-driven approach, while incorporating scalable, minimally invasive, valid, measurement methods such as actigraphy (or validated accelerometry with low-power settings) conducted over an extended period of time. Additionally, brief parent- and teacher-reported assessments of daytime functioning—including measures of inattention, mood, and sleepiness—should be collected, alongside school attendance and malaria episode information sourced from existing registries. Within the 7–11 year age range, early indicators of risk are likely to emerge in cognitive domains such as executive function, reading and mathematics fluency, and behavioral regulation [[3], [4], [5], [6], [7]]. Collaborative efforts with ministries of education can facilitate classroom-based assessments while minimizing disruption to families. Furthermore, local production of mattresses and nets, the establishment of repair and retreatment systems, and the development of community micro-enterprises focused on bedding may enhance both the sustainability of programmes and household engagement [2].

The Tanzanian experience offers a concise framework for “micro-sleep equity”: (1) ensure vector barriers and thermal comfort suitable to local conditions; (2) use schools for identifying, distributing, and following up; (3) provide brief, practical sleep education for caregivers on routines and environment; and (4) implement context-sensitive measurement to determine intervention impact on sleep and daytime functioning. These steps require neither specialized clinics nor expensive devices and support community engagement and supportive environments [1,14].

## 2 Conclusion

To improve children's sleep health across entire populations, solutions need to go beyond just laboratory work or top-down policies. Real progress happens when we make small, everyday spaces safer and more consistent—one family and one school at a time. The bed-kit program in Tanzania demonstrates that creating supportive micro-environments can be effective, approachable, and expanded to wider use—and may even aid child development [2]. Instead of waiting, it's important to put these ideas into action and measure results as communities take the lead, allowing science to evolve alongside them [13,15].

## Declaration of competing interest

The authors declare that they have no known competing financial interests or personal relationships that could have appeared to influence the work reported in this paper.
